# Proximity-dependent biotin identification (BioID) reveals a dynamic LSD1–CoREST interactome during embryonic stem cell differentiation†

**DOI:** 10.1039/d1mo00236h

**Published:** 2021-10-14

**Authors:** Claire E. Barnes, David M. English, Megan Broderick, Mark O. Collins, Shaun M. Cowley

**Affiliations:** Department of Molecular and Cell biology, University of Leicester Henry Wellcome Building Leicester LE1 7RH UK Smc57@le.ac.uk +44 (0)116 2297098; School of Biosciences, University of Sheffield Western Bank Sheffield S10 2TN UK; Faculty of Science Mass Spectrometry Centre, University of Sheffield Brook Hill Road Sheffield S3 7HF UK

## Abstract

Lysine specific demethylase 1 (LSD1) regulates gene expression as part of the CoREST complex, along with co-repressor of REST (CoREST) and histone deacetylase 1 (HDAC1). CoREST is recruited to specific genomic loci by core components and numerous transient interactions with chromatin-associated factors and transcription factors. We hypothesise that many of these weaker and transient associations may be difficult to identify using traditional co-immunoprecipitation methods. We have therefore employed proximity-dependent biotin-identification (BioID) with four different members of the CoREST complex, in three different cell types, to identify a comprehensive network of LSD1/CoREST associated proteins. In HEK293T cells, we identified 302 CoREST-associated proteins. Among this group were 16 of 18 known CoREST components and numerous novel associations, including readers (CHD3, 4, 6, 7 and 8), writers (KMT2B and KMT2D) and erasers (KDM2B) of histone methylation. However, components of other HDAC1 containing complexes (*e.g.* Sin3) were largely absent. To examine the dynamic nature of the CoREST interactome in a primary cell type, we replaced endogenous LSD1 with BirA*-LSD1 in embryonic stem (ES) cells and performed BioID in pluripotent, early- and late-differentiating environments. We identified 156 LSD1-associated proteins of which 67 were constitutively associated across all three time-points (43%), including novel associations with the MMB and ChAHP complexes, implying that the majority of interactors are both dynamic and cell type dependent. In total, we have performed 16 independent BioID experiments for LSD1 in three different cell types, producing a definitive network of LSD1-assoicated proteins that should provide a major resource for the field.

## Abstract 2

BIO-ID identifies different subsets of proteins associated with the LSD1-CoREST complex during stem cell differentiation.

## Graphical abstract

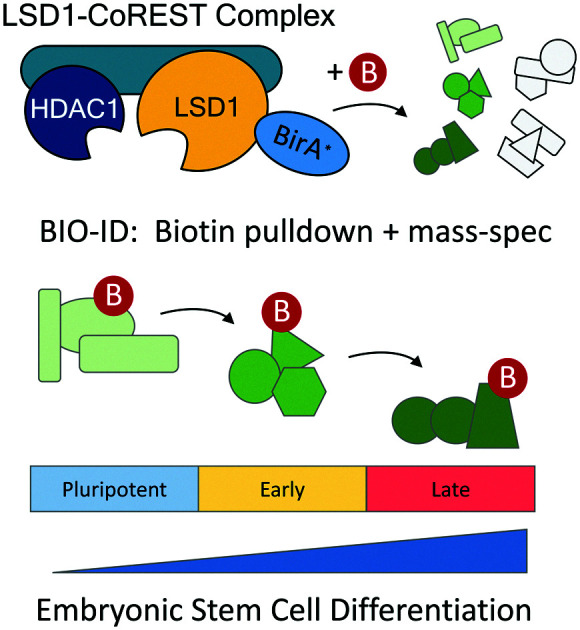

## Introduction

The ability to regulate chromatin structure is a critical step in determining patterns of gene expression in eukaryotic cells. The core histones (H2A, H2B, H3 and H4) are subject to a myriad of post-translational modifications (PTM) that alter their chemical properties and act as binding sites for the recruitment of additional proteins. Lysine acetylation (Lys-Ac) is a common PTM in histones, occurring primarily on the Lys rich N-terminal tails of histones.^1^ Addition of the acetyl-moiety neutralizes the positive charge on the Lys, reducing the association of the histone tail with the DNA backbone and negative patches on neighbouring nucleosomes. Lys-Ac is a dynamic modification whose levels are determined by the competing activities of histone acetyl transferases (HATs) and histone deacetylases (HDACs). Among the 18 HDACs found in mammalian cells the highly related enzymes, HDAC1 and HDAC2 (HDAC1/2), contribute approximately half of the total deacetylase activity,^2,3^ as part of four canonical multi-protein complexes: Sin3, NuRD, CoREST and MiDAC.^4,5^ Each complex typically contains 3–6 core proteins with a coterie of additional subunits thought to add functionality by directing genomic location and substrate specificity. There is relatively little overlap in terms of protein components between the complexes and knockout studies suggest that each of the four has a unique function during development.^4^ Given the multitude of protein–protein interactions present within each of these complexes, a number of molecular details still remain to be addressed. In particular, the identity and number of complex components present at any given time need to be elucidated. Indeed, there is evidence to suggest that HDAC1/2 complexes are able to adapt their auxiliary components to suit different cell types.^6^

The core CoREST complex is comprised of three components: lysine specific demethylase 1 (LSD1/KDM1A), co-repressor of RE1-silencing transcription factor (CoREST) and HDAC1. LSD1 was the first protein demethylase to be identified and acts to remove the mono- and di-methyl moieties from Lys4 on histone H3 (H3K4me/me2),^7–10^ a positive mark in regards to transcriptional activity, although it potentially has additional histone substrates.^11,12^ The amine oxidase domain of LSD1 contains a long helical ‘Tower’ domain which forms a heterodimer with CoREST.^13–16^ CoREST binding stimulates LSD1 demethylase activity,^9,17^ it also contains an N-terminal ELM2/SANT domain that binds to HDAC1^18,19^ and a second SANT domain in the C-terminus. The combination of demethylase/deacetylase activities within the core LSD1/CoREST/HDAC1 complex allows for the coordinated demethylation of H3K4me/me2 and deacetylation of the histone H3 tail, promoting transcriptional repression.^9,16,17,20,21^ Indeed, *in vitro* studies with purified LSD1/CoREST and cellular experiments suggest that the deacetylated H3 tail may be the preferred LSD1 substrate for demethylation.^17,20^ In addition to the core components, the CoREST complex contains a number of additional chromatin associated subunits, such as PHF21A, HMG20A/B and CTBP1/2 which enable recruitment to specific genomic loci.^7,9^

The association of LSD1/CoREST with both core components and accessory proteins is critical to their function in cells. While the majority of LSD1-interacting proteins have been identified by co-immunoprecipitation methods, we hypothesise that this will likely miss many transiently associated proteins, including substrates of LSD1 and HDAC1. We have therefore utilised a proximity-dependent biotin identification (BioID) approach. BioID utilizes a biotin ligase fused to the protein of interest such that proximal proteins (∼10 nm) are labelled with biotin, which can then be identified by streptavidin pull-down followed by quantitative mass-spectrometry.^22,23^ The advantage of the BioID approach is that unlike co-purification methods, it does not require direct binding and thus enables identification of transiently associated proteins, or those in close proximity. To increase the confidence of our experiments we tagged four separate components of the CoREST complex (LSD1, CoREST1, HDAC1 and PHF21A) with the biotin ligase, BirA* and identified an extensive protein network in HEK293T and embryonic stem (ES) cells. Retaining only targets identified with 3 out of 4 bait proteins, we identified 302 CoREST-associated proteins in HEK293T cells. Among this group were 16 of 18 known CoREST components and numerous novel associations, including readers (CHD3, 4, 6, 7 and 8), writers (KMT2B and KMT2D) and erasers (KDM2B) of histone methylation. However, components of other HDAC1 containing complexes (*e.g.* Sin3A, NuRD) were largely absent, suggesting that CoREST functions in a chromatin based environment that is independent of these related complexes. To examine the role of CoREST in different cell types, we replaced endogenous LSD1 with BirA*-LSD1 in embryonic stem (ES) cells and performed BioID in pluripotent, early- and late-differentiating environments. In total, we identified 156 LSD1-associated proteins of which 67 were constitutively associated across all three time-points (43%), including novel associations with the MMB and ChAHP complexes. These data imply that the majority of CoREST interactions (56%) are dynamic and highly dependent on cell type. Furthermore, similar to HEK293T cell data, the LSD1 interactome in embryonic stem (ES) cells was lacking components of other HDAC1-containing complexes. Our data suggests that CoREST occupies a discrete space in the nucleus with a specific subset of transcription factors and chromatin modifiers that appears to be largely distinct from other HDAC1-containing complexes. Our application of BioID has identified a definitive dataset of LSD1 associated proteins and novel interactomes for CoREST1 and PHF21A, that should be of wide utility to the field.

## Results

### Efficient labelling and identification of LSD1/CoREST/HDAC1 proximal proteins using BioID

**Fig. 1:**
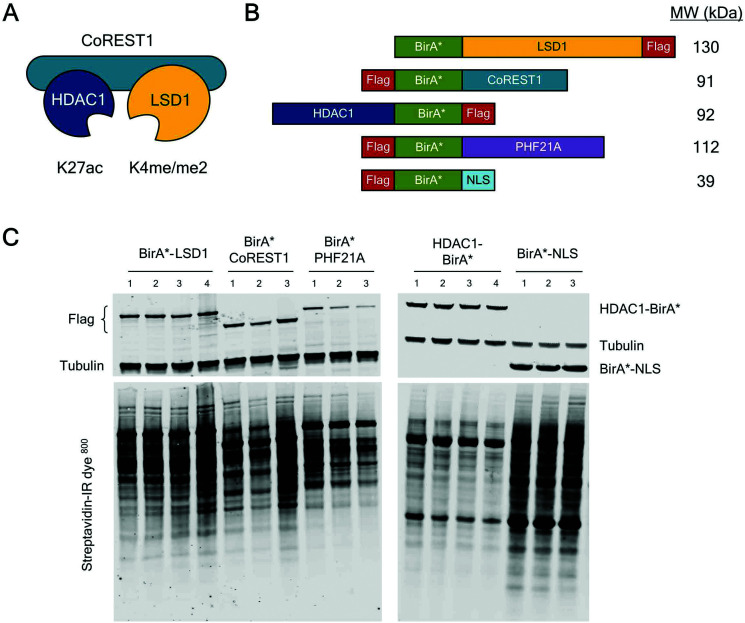
CoREST complex members tagged with BirA* expressed in HEK293T cells biotinylated proximal proteins. (A) Schematic diagram showing the ternary CoREST complex (LSD1/CoREST1/HDAC1), their preferred substrates in histone H3 and mode of assembly. (B) Schematic diagram of the BirA*-tagged proteins used in the study. The relative position of BirA* and FLAG-tags are indicated. (C) Anti-FLAG and anti-Tubulin antibodies were used (on the same blot) to test the relative expression of BirA*-tagged proteins (upper panel) in HEK293T cells. Proteins biotinylated by the indicated constructs were detected by probing western blot membranes with streptavidin-IR^800^. Each lane represents an individual biological replicate used in the BioID study.

The core ternary CoREST complex (Fig. 1A) is recruited to specific genomic loci by co-factors with histone recognition motifs (*e.g.* PHF21A, PHD domain^9^) and transcription factors, such as REST.^24^ In addition, LSD1 and HDAC1 are both enzymes, whose range of histone and non-histone substrates are likely to be transiently associated. Therefore, an understanding of the local protein environment around the CoREST complex will be instrumental in defining its role in cells at a molecular level. To this end, we employed BioID methodology, which utilizes a promiscuous version of the *E. coli* biotin ligase enzyme, BirA (BirA*-R118G^22,23^), fused to the protein of interest, allowing labelling and identification of proximal proteins (within approx. 10 nm). The advantage of this approach is that it allows the identification of both binding partners and transiently associated proteins such as substrates. To investigate the proximal protein environment of the CoREST complex five BirA* fusion protein constructs were generated (Fig. 1B). These included the three core protein components, LSD1, CoREST1 and HDAC1 (Fig. 1A); and PHF21A which forms part of an extended complex, originally termed the BRAF–HDAC complex (BHC).^7^ As a control, the BirA* protein was tagged with a nucleoporin nuclear localisation signal (NLS, BirA*-NLS), to identify proteins incidentally biotinylated by BirA* alone. Due to the presence of unstructured N-terminal regions in LSD1, PHF21A and CoREST1, the BirA* tag was added to their N-termini, whereas HDAC1 was C-terminally fused to BirA* because the N-terminus forms part of the histone deacetylase domain.^18^ In addition, all the constructs contained a FLAG-tag to examine their relative expression levels in cells. Transient transfection of HEK293T cells followed by western blotting revealed that each of the constructs was expressed (Fig. 1C) and that BirA*-LSD1 and HDAC1-BirA* levels approximated those of the endogenous protein (Fig. S1A, ESI†). Addition of biotin to the media of these transfected cells caused efficient biotinylation of a range of proteins of various molecular weights, as visualized using a streptavidin-IR dye conjugate (Fig. 1C and Fig. S1B, ESI†). The overall intensity of biotin labelling appeared to be consistent across technical and biological replicates, and between individual CoREST complex members. However, it was noticeable that the BirA*-NLS control consistently produced higher levels of biotinylation in comparison to CoREST components. This may be due to higher protein expression levels, given the smaller size of the BirA*-NLS construct, or the absence of a nuclear function leading to greater promiscuity of associated proteins.

**Fig. 2:**
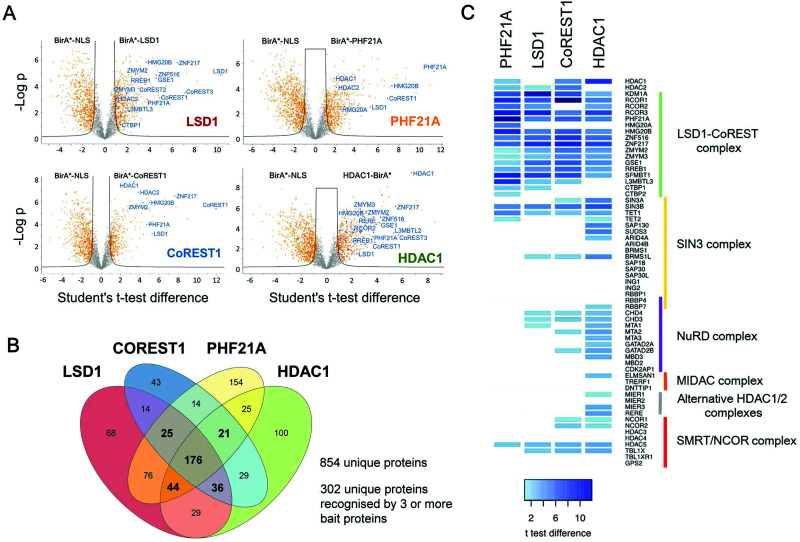
Identification of CoREST complex proximal proteins. (A) Volcano plots of LFQ data from BirA-tagged bait proteins *versus* the BirA-NLS control are shown. Two levels of stringency for the Student's *t*-test were utilised based on the *S*0 parameter in Perseus which controls the relative importance of a *t*-test *p* value and difference between the means; *S*0 = 1, a lower stringency level (orange squares), and *S*0 = 2 a higher stringency level (solid black lines). All subsequent analysis was performed on proteins significantly enriched in the higher stringency analysis. Previously known interacting proteins (as determined from BioGRID^67^) are highlighted (blue squares) and components of the CoREST complex labelled for reference. (B) Significantly enriched proteins for each BirA*-fused CoREST complex member were determined by students *t*-tests at high stringency (*S*0 = 2). The proximal proteins for each CoREST complex component were compared and shown to overlap substantially. (C) A heat-map was plotted based on the students *t*-test differences (*vs.* BirA*-NLS) for association of class-I HDAC complexes components with the indicated CoREST components.

To assess the local protein environment of the CoREST complex protein each construct was transfected independently into HEK293T cells in triplicate (BirA*-NLS, BirA*-PHF21A, BirA*-CoREST1) or quadruplicate (BirA*-LSD1, HDAC1-BirA*). Biotinylated proteins were isolated from cell lysates, sonicated for 4 min to release chromatin associated proteins (Fig. S1C, ESI†), using streptavidin coated beads, trypsin digested and analysed by quantitative tandem mass spectrometry. Peptide and protein identification, label free quantitation (LFQ) and initial data analysis were performed to identify proteins biotinylated in multiple replicates of BioID with a single BirA*-fused protein. After this initial processing, a total of 3103 proteins were identified. Following quality control, normalisation and imputation of missing values, students *t*-tests were performed for each CoREST complex member to determine the proteins significantly enriched compared to the BirA*-NLS control sets. The volcano plots (Fig. 2A) show data for individually identified proteins for each of the CoREST complex members used in the assay. BirA*-LSD1 specifically labelled each of its potential dimeric partners CoREST1, 2 and 3. Members of the extended CoREST complex identified in previous studies, PHF21A, HMG20B (BRAF35), RREB1 and GSE1 were also among the most significantly enriched proteins. This indicates that proteins which form part of the constitutive CoREST complex are more likely to be identified using BioID. Interestingly, HDAC1, which forms part of the core ternary CoREST complex, was only weakly associated with LSD1, suggesting that the position of BirA* (N- *vs.* C-terminus) and the degree of freedom from the bait protein are important determinants for labelling. HDAC1 is known to be present in a number of different histone modifying complexes, including Sin3, NuRD and MiDAC.^4,5^ Consistent with this, we were able to identify numerous components of these complexes, Sin3A/B, MTA1/2/3 and ELMSAN1. To examine whether components of the CoREST complex overlap with members of other HDAC1/2 containing complexes, we plotted the CoREST-proximal proteins against the known components of the other class-I HDAC containing complexes (Fig. 2C). We find that there is relatively little overlap in terms of shared components between CoREST and other complexes, with only HDAC1 consistently associating with multiple complexes as would be expected. This is significant, because it suggests that LSD1/CoREST operates in a chromatin environment that is independent of other HDAC1 containing complexes (Sin3A, NuRD and MiDAC).

### BioID identifies the majority of known CoREST associated proteins and significantly extends the list of potential cooperating factors

**Fig. 3:**
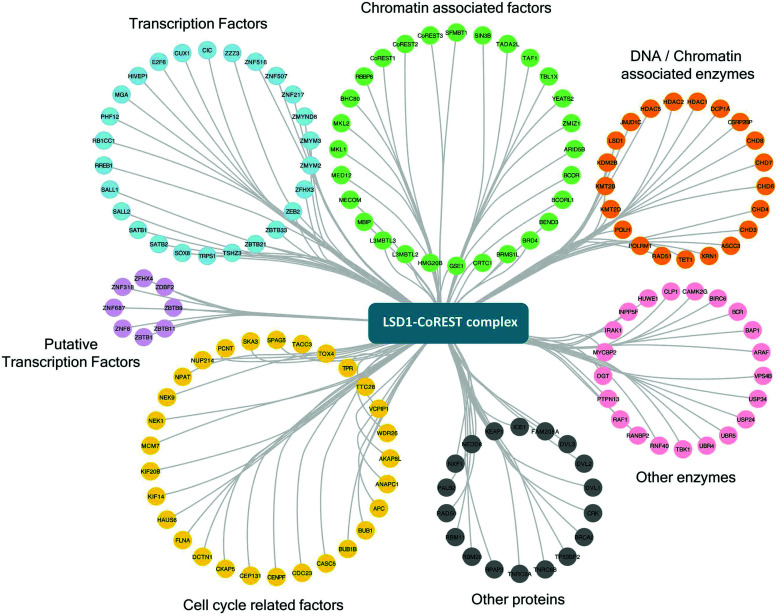
CoREST associated proteins and their biological functions. Proteins significantly associated with three or more components of the CoREST complex (*S*0 = 2) using BioID, were categorized by their individual functions using ClueGO^68^ and mapped in Cytoscape.^54^

One of the strengths of our study is that we were able to test multiple components of the same complex individually and therefore have increased confidence in the targets identified. To generate a definitive picture of the proximal protein environment surrounding the CoREST complex we applied a stringent cutoff to our dataset that only proteins that were significantly enriched with 3 out of 4 bait proteins would qualify as CoREST-associated (Fig. 2B and Table T1, ESI†). While strict, this still allows some leeway for the position of BirA* within the complex and its accessibility for labelling nearby proteins. Application of these criteria gives us a list of 302 out of 854 proteins (35% of total), which includes almost all known CoREST complex components (Fig. 2C, 16/18 proteins), with the exception of CTBP1 and 2, which are only labelled by BirA*-PHF21A. This is a high degree of overlap considering that HDAC1 has 100 unique targets in separate class-I HDAC complexes (Fig. 2C). We hypothesize that this list of 302 proteins contains a number of factors which cooperate functionally with the CoREST complex, including histone modifying enzymes, histone binding proteins and transcription factors. We would also expect there to be substrates of both LSD1 and HDAC1 among this group since the signal from transient protein–protein interactions would accumulate over the 18 hour biotinylation period. As shown in Fig. 3, we observed a significant association of both chromatin modifying and chromatin associated factors. This included the H3K4 methyltransferases, KMT2B (MLL2) and KMT2D (MLL4), which methylate distinct regions of the genome as part of discrete multi-subunit complexes.^25^ The proximity of these methyltransferases suggests a degree of overlap in the methylation/demethylation of H3K4, as observed during transcriptional cycling.^26^ We also observed an association of the CoREST complex with two non-canonical polycomb repressive complexes, PRC1.1 and PRC1.6. KDM2B is a lysine demethylase which can be targeted to unmethylated CpG islands through its CXXC domain, and along with BCOR, helps PRC1.1 maintain the integrity of polycomb domains throughout the genome.^27^ KDM2B demethylates H3K4me3,^28^ while LSD1 can only demethylate H3K4me/me2, which highlights the possibility for synergy between CoREST and PRC1.1 complexes in removing this modification of transcriptionally active chromatin. We also identified E2F6, L3MTBL2 and MGA, three components of PRC1.6, which is required for the repression of meiotic and germ-line-specific genes.^29^ In addition to histone writers, there were a number of methyl-lysine readers, including the chromodomain containing proteins, CHD3, 4, 6, 7 and 8, which fit the role of LSD1 as a regulator of histone methylation. There were also 26 different transcription factors, including previously known CoREST components (ZNF516, ZNF217, ZMYM2 and ZMYM3) and novel associations, such as SATB1 (and its homolog, SATB2), which, when phosphorylated, associates with HDAC1 and causes transcriptional repression.^30^ Unexpectedly, we also identified a significant number of cell cycle related factors, in particular, components and regulators of the anaphase promoting complex/cyclosome (APC/C): ANAPC1, CDC23, BUB1 and BUB1B.^31^ Collectively, these data indicate the CoREST complex occupies a discrete space within the nucleus with a specific subset of transcription factors and chromatin modifiers, and importantly, these appear to be largely independent of other class-I HDAC complexes.

### Identification of CoREST associated proteins during ES cell differentiation

**Fig. 4:**
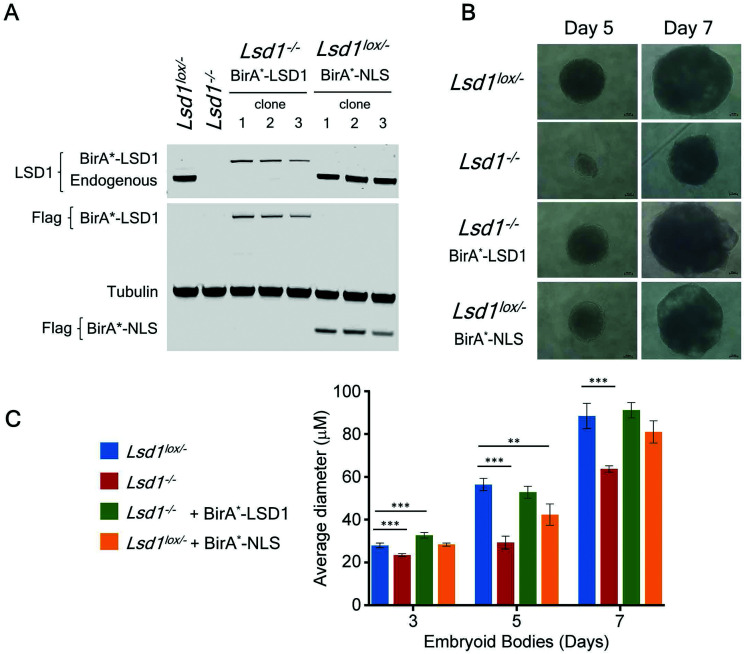
BirA*-LSD1 expression rescues the loss of function phenotype in *Lsd1-knockout* (*Lsd1*^*−/−*^) ES cells. A, western blot showing endogenous LSD1, or BirA*-LSD1 in *Lsd1*^*−/−*^ and *Lsd1*^*Lox/−*^ ES cells, using anti-FLAG, or anti-LSD1 antibodies as indicated. B, Representative images of embryoid bodies (EBs) at day 5 and day 7 of differentiation from the ES cell lines as indicated. C, Average EB diameter over a 7-day time course. All values are shown as mean ± SD. All data points were assessed for statistical significance relative to control cells (*Lsd1*^*Lox/−*^) using a student's *t*-test (*p* ≤ 0.05 = *, *p* ≤ 0.01 = **, *p* ≤ 0.001 = ***). All significant values are shown.

Many of the major proteomic studies of the CoREST complex have used either HEK293T^9^ or HeLa cell lines.^7,8,17^ While useful biochemical tools, these are transformed cells that do not faithfully model the physiological and developmental roles of CoREST *in vivo*. *Lsd1* is an essential gene in mice, with embryonic lethality occurring prior to gastrulation at e6.5 days.^32–34^ Embryonic stem (ES) cells are a model for cells of the inner cell mass (e3.5 days) and are able to proliferate normally in the absence of LSD1. However, *Lsd1* null ES cells are unable to differentiate properly, showing mis-regulation of major differentiation pathways and elevated levels of cells death.^32,33^ We took advantage of *Lsd1* conditional knockout (cKO) ES cells to stably reintroduce our BirA*-LSD1 construct, such that it was the only LSD1 protein being expressed (Fig. 4A). In parallel, we also generated BirA*-NLS control cells using the same parental ES cell line. Western blotting for LSD1 showed that BirA*-LSD1 is expressed at a lower level than the endogenous LSD1 protein. However, upon differentiation to embryoid bodies, we found that BirA*-LSD1 was able to rescue the growth and differentiation defects observed in *Lsd1-KO* cells (Fig. 4B, C and ref. 32), confirming that the fusion protein is functional and capable of replacing endogenous LSD1. We have thus generated a flexible primary cell system in which to examine the proximal protein environment of LSD1.

**Fig. 5:**
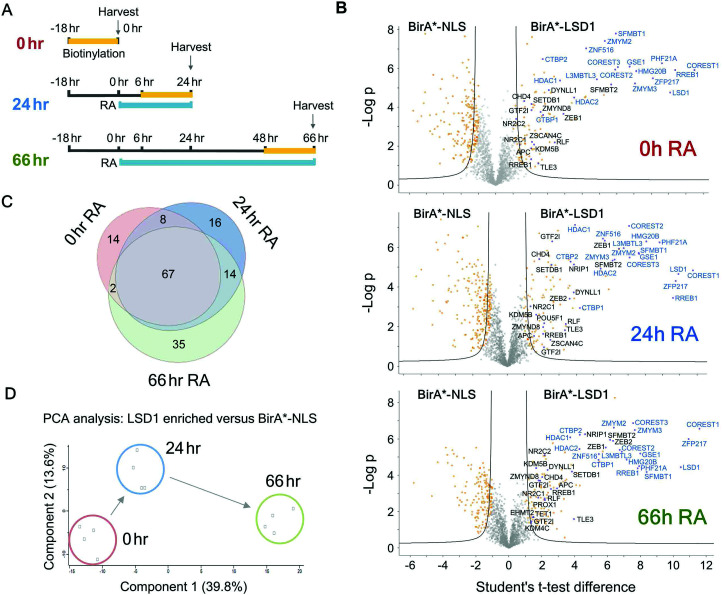
ES cells were differentiated with retinoic acid (RA) to identify the temporal LSD1 interactome during differentiation. (A) Schematic diagram showing the time course for RA treatment and biotin labelling used for BioID experiments. (B) Volcano plots for the indicated time points of RA treatment. Two levels of stringency for the Student's *t*-test were utilised based on the *S*0 parameter in Perseus which controls the relative importance of a *t*-test *p* value and difference between the means; *S*0 = 1, a lower stringency level (orange squares), and *S*0 = 2 a higher stringency level (solid black lines). All subsequent analysis was performed on proteins significantly enriched in the higher stringency analysis. Previously known interacting proteins (as determined from BioGRID^67^) are highlighted (blue squares) and components of the CoREST complex labelled for reference. (C) Venn diagram showing proteins significantly associated with BirA*-LSD1 (*S*0 = 2) at the indicated time-points following RA treatment. (D) Principal component analysis (PCA) was performed on individual BirA*-LSD1 BioID experiments (*n* = 4) at the indicated time-points.

In addition to the role of the LSD1 in pluripotent ES cells, we wanted to explore the changing proteomic environment of the CoREST complex in actively differentiating cells. We hypothesised that LSD1 would likely exchange its coterie of associated proteins as cells transitioned from one lineage to another. To test this, we treated BirA*-NLS and BirA*-LSD1 ES cells with retinoic acid (RA) to stimulate ES cell differentiation and generated biotin labelled extracts at 0, 24 and 66 hours time points (Fig. 5A). To confirm that the cells were undergoing differentiation we performed western blotting for stem cell (OCT4 and NANOG) and lineage specific (GATA4) markers (Fig. S2, ESI†). As expected, both OCT4 and NANOG levels decreased over time and were absent by the 66 hours time point, while GATA4 expression was increased. Samples were then processed for BioID in an identical manner to the previous experiments in HEK293T cells, with LSD1-proximal proteins identified by mass-spectrometry and those showing significant quantitative enrichment over BirA*-NLS plotted for each time point (Fig. 5B). In total we identified 156 individual LSD1-associated proteins across the time course (Fig. 5C and Table T2, ESI†). Immediately apparent is that all of the core CoREST complex members (18 out of 18) are among the 67 proteins which remain stably associated during differentiation (Fig. 5C), with CoREST1/2/3, HDAC1/2, PHF21A, GSE1 and ZMYM2/3 among the most significantly enriched proteins identified in all samples. However, we also identified proteins whose association is specific to pluripotent cells (RA 0 h, 14 proteins), early differentiation (RA 24 h, 16 proteins) and differentiated cells (RA 66 h, 35 proteins). Indeed, principal component analysis (PCA) of LSD1 proximal proteins at the three different time points (using four independent BirA*-LSD1 clones) reflected a transition in CoREST environment during the process of differentiation (Fig. 5D). To some extent, this may reflect the changes in gene expression during differentiation, typified by the association with the co-repressor, NRIP1 (24/66 hours), whose expression is induced by RA treatment. However, the expression of most differentiation-specific targets (CBX3, EHMT2, JARID1C, TFDP1, *etc.*) are unaffected by differentiation and therefore represents a distinct set of associated proteins during the ES to somatic cell transition. Intriguingly, among this group is the stem cell master regulator, OCT4 (RA 24 h only). OCT4 has a pro-differentiation role in early embryogenesis^35,36^ and has previously been demonstrated to bind LSD1 containing complexes,^37–39^ which our data suggest may be induced by differentiation.

### The LSD1/CoREST interactome in ES cells is enriched from chromatin modifying complexes

**Fig. 6:**
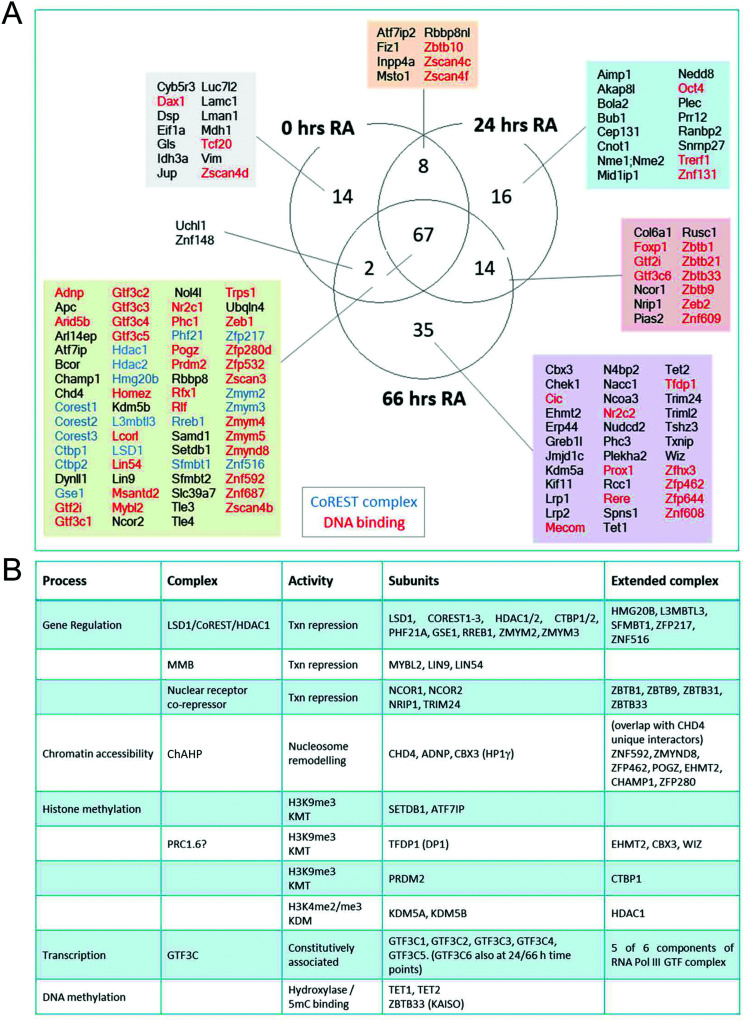
LSD1–CoREST has a dynamic complexome during ES cell differentiation. (A) Venn diagram showing number and names of proteins significantly associated with BirA*-LSD1 (students *t*-test, *S*0 = 2) at the indicated time-points following RA treatment. (B) Table showing LSD1 associated chromatin complexes, subunits identified with BirA*-LSD1, their extended complex components and biological activities.

The advantage of the BioID approach is that unlike co-purification methods, it does not require direct binding. We detected a number of protein complexes with chromatin modifying activities that presumably are proximal to the CoREST complex and whose activity is complimentary to its demethylase/deacetylase activity. Two core components of the MuvB complex (LIN9 and LIN54), as well as the associated protein MYBL2, were identified at all 3 time points (Fig. 6A). The MuvB complex plays dual roles in regulating the cell cycle. During G0 and early G1 MuvB forms part of the DREAM complex which represses G1/S and G2/M phase genes.^40,41^ While during late G1 MuvB dissociates from the DREAM complex and associates with MYBL2, forming the Myb-MuvB (MMB) complex,^42^ which stimulates the expression of genes that are important for G2/M phases.^43^ We also detected potentially cooperating methyltransferases and demethylases. KDM5A (0/24/66 hours) and KDM5B (66 hours) both remove H3K4me3.^44,45^ KDM5B regulates H3K4 methylation during ES cell differentiation and acts in concert with LSD1 to regulate H3K4 methylation at highly active ES cell genes.^46^ This lends credence to the idea of cooperation between pairs of demethylases leading to complete H3K4 demethylation in ES cells, as is the case for LSD1 and KDM5B in breast cancer cells.^47^

Consistent with CoRESTs role as a transcriptional repressor, we also identified an H3K9me3 methyltransferase, SETDB1, and its binding partner ATF7IP (both proteins identified at all time points). ATF7IP is proposed to stimulate SETDB1 activity by promoting its nuclear import and preventing its degradation.^48,49^ Among the differentiation-specific proximal proteins were CBX3, EHMT2 (G9a) and TFDP1, components of the non-canonical PRC1 complex, PRC1.6/E2F6.com.^29^ E2F6 was not present in our ES cells experiments but was identified by all of the bait proteins used in HEK293T cells (Fig. 3). EHMT2 is a KMT for H3K9me2, suggesting that the application of this heterochromatic mark may be coordinated with the removal of H3K4me/me2 by LSD1. EHMT2 has also been shown to methylate the N-terminus of LSD1 at K114, a mark which is recognized by the chromodomain of CHD1,^50^ indicating that in addition to identifying LSD1 substrates, BioID can identify enzymes which also modify LSD1. Unexpectedly, the 5-methylcytosine hydroxylases, TET1 and TET2, were identified as differentiation specific CoREST-proximal proteins (Fig. 6). LSD1 activity has been linked to DNA methylation through the stabilization of DNMT1.^33^ Although a potential substrate of LSD1, DNMT1 was not present in our BioID data from either ES or HEK293T cells. The identification of both TET1 and TET2 (TET3 is not expressed in ES or somatic cells) and 5-methyl-CpG binding factor, ZBTB33 (aka KAISO) all lend credence to the association of H3K4me/me2 demethylation and DNA methylation activities (Fig. 6B) that will require further investigation. As in HEK293T cells (Fig. 2C), we again failed to identify components of other HDAC1-containing complexes (Sin3A, NuRD and MiDAC) in ES cells using BirA*-LSD1, which supports the notion that LSD1 operates in chromatin environment which is exclusive to CoREST.

We found a consistent association with the ATPase/chromatin remodelling protein, CHD4. CHD4 forms part of the nucleosome and remodelling and deacetylase (NuRD) complex^51^ that has been proposed to also contain LSD1.^52^ However, we find no association of LSD1 with NuRD subunits (MTA1-3, MBD3, GATAD2A) in ES cells (Fig. 6). More recently, CHD4 was found to form a complex with ADNP (activity dependent neuroprotective protein) and heterochromatin protein 1 gamma (HP1γ, aka CBX3), called ChAHP, which negatively regulates chromatin accessibility.^53^ Intriguingly, we detect ADNP, CHD4 and CBX3 with BirA*-LSD1 in ES cells; and observe a significant overlap between unique CHD4 interactors^53^ and LSD1-associated proteins (ZNF592, ZMYND8, ZFP462, POGZ, EHMT2, CHAMP1, ZFP280), suggesting that CoREST and ChAHP complexes may cooperate to regulate chromatin accessibility.

**Fig. 7:**
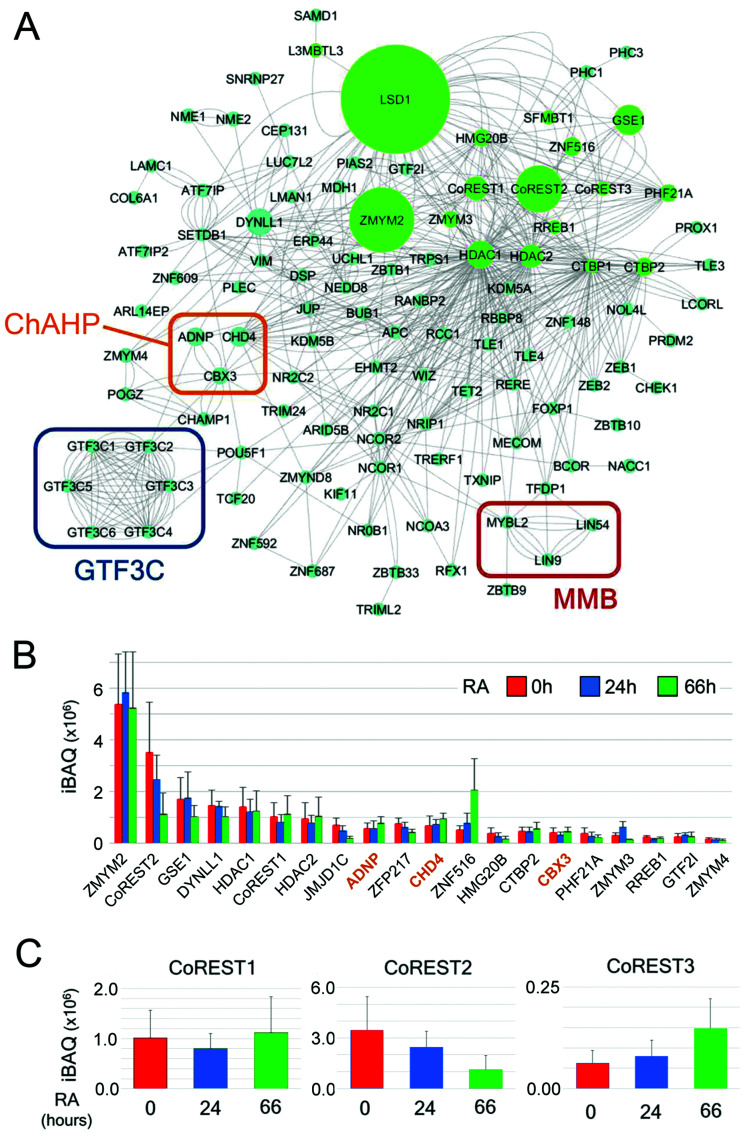
LSD1-interactome network and iBAQ values reveal shifting stoichiometry of the CoREST complex. (A) Cytoscape was used to generate a network of LSD1-interacting proteins in ES cells. The intensity-based absolute quantification (iBAQ) values for individually isolated proteins is indicated by the size of the node (green circles). LSD1-associated complexes of interest, including: MMB, ChAHP and GTF3C are indicated by coloured rectangles. (B) The top 20 proteins with the highest iBAQ values are shown for each time-point of retinoic acid (RA) treatment. The three main components of the ChAHP complex are coloured in orange. All values are shown as mean ± SD (C) iBAQ values for the three CoREST homologues is shown over the time course of RA mediated differentiation.

Finally, we used Cytoscape^54^ to plot a network of LSD1 interacting proteins based on known associations in combination with intensity-based absolute quantification (iBAQ) values for individual proteins (Fig. 7A). iBAQ values derived from BioID give an indication of both the abundance of proteins within a sample and their proximity to the N-terminus of LSD1 for labelling. This network identifies the presence of potential cooperating complexes, such as MMB and ChAHP (discussed above), and intriguingly, 6 of 6 components of the RNA pol III general transcription factor 3C (GTF3C). GTF3C binds to the internal promoters of the majority of RNA pol III genes (including tRNAs) and helps recruit GTF3B for transcription initiation.^55^ The presence of all six GTF3C subunits is compelling and suggests an unrecognised role for the CoREST complex in regulating tRNA transcription. The iBAQ values derived from LSD1-associated proteins also give an indication of their stoichiometry within the complex and proximity to CoREST. Unsurprisingly, 14 of 20 proteins with the highest iBAQ values are components of the CoREST complex itself (Fig. 7B). All 3 members of the ChAHP complex (CHD4, ADNP and CBX3) are among this group, reiterating the potential association of this complex with CoREST. iBAQ values across the time-course of RA treatment also allows us to identify changes in stoichiometry of LSD1-associated proteins. We detect a 4-fold increase in ZNF516 association for instance, which correlates with increased levels of *Znf516* transcription during ES cell differentiation. LSD1 has three potential heterodimeric partners, CoREST1-3. CoREST2 is the major partner in pluripotent ES cells (Fig. 7B), but decreases during differentiation to levels equivalent to CoREST1. CoREST3 is by far the junior partner among the three proteins, although it shows increased binding to LSD1 following RA treatment (Fig. 7C). Collectively, our data shows subtle changes within the CoREST complex during differentiation, and a chromatin environment composed of constitutively associated complexes (MMB, GTF3C, SETDB1/ATF7IP) and a changing set of partners (*e.g.* TET1/TET2) reflecting the alteration in cellular context.

## Discussion

Understanding the extended ‘social network’ of a protein (binding partners, substrates, modifying enzymes, complex subunits, *etc.*) is crucial to understanding its molecular mechanism in cells. Historically, proteomic approaches have taken advantage of yeast two-hybrid, or affinity-purification coupled to mass-spectrometry, to identify candidate lists of associated proteins. The latter relies on protein partners remaining bound throughout cell lysis and then subsequent wash-steps to allow for their identification. While this technique has been hugely powerful^56,57^ it potentially misses many weak or transient associations that occur throughout a dynamic proteome. Here we have explored the interactome of the protein demethylase, LSD1, using BioID. BioID covalently attaches biotin to protein partners within a range of ∼10 nm allowing transient associations to be captured.^22,23,58^ To increase the confidence of protein partners identified, we performed BioID for all of the core components of the CoREST complex: LSD1, CoREST1, HDAC1 and PHF21A. In each case, the most enriched proteins were components of the CoREST complex (Fig. 2A and C), validating the assay and also indicating that stable complex components are the proteins most likely to be identified. To produce a definitive set of CoREST associated factors in HEK293T cells we applied a stringent cut-off, that potential partners must be enriched with 3 out 4 bait proteins, which narrowed the list to 302 candidate proteins, including numerous transcription factors and chromatin associated factors (Fig. 2 and 3). Among this group were 16 of 18 core CoREST complexes members (CTBP1 was only found with BirA-PHF21A), that gives us confidence in the extended list of previously unidentified partners. For instance, we find a strong overlap with the CoREST complex and histone methylation, including readers (CHD3, 4, 6, 7 and 8), writers (KMT2B and KMT2D) and erasers (KDM2B), suggesting functional cooperativity in the regulation of H3K4 and H3K9 methylation. PRC2 complex components (which regulates H3K27 methylation) were absent from our survey, but we did find PRC1.1 (KDM2B, BCOR) and PRC1.6 (E2F6, L3MTBL2, MGA), which indicates functional overlap between complexes that repress transcription.

Given these data, it is worth considering the practical labelling distance of BirA* which has been previously determined at ∼10 nm (∼100 Å).^58^ While this distance is biologically relevant for studying the CoREST complex, LSD1 has an elongated structure ∼150 Å in length^15^ and a nucleosome octamer is ∼65 Å in length,^59^ suggesting that the physical arrangement of proteins in a complex might be a factor for biotin-labelling. If so, then BioID may identify only a subset of components within a given multi-subunit complex, while preferentially labelling others (*e.g.* ZMYM2, Fig. 7). Our data from LSD1-associated complexes suggests this might be the case. BirA*-LSD1 identified three components of the MMB complex in ES cells, MYBL2/LIN9/LIN54, but not extended complex members such as RBBP4/LIN37/LIN52 (Fig. 6A and B), suggesting that only the former proteins are located physically in proximity to LSD1. One of the strengths of BioID is that it has the capacity to identify weak and transient associations, including, enzyme substrates. We did not detect either p53 or DNMT1, both known substrates of LSD1,^33,60^ in our experiments, although both proteins are expressed in HEK293T and ES cells. However, we did detect an association between LSD1 and KIF11, a kinesin-like protein whose ATPase activity is regulated by Lys-acetylation and is a substrate for HDAC1.^61^

## Conclusions

In total, we have performed 16 independent BioID experiments for LSD1 in three different cell types (ES, ES + RA and HEK293T). Interestingly, only 43 out of 577 proximal proteins (7% of total) were identified under all conditions (Table T3, ESI†), which underlines the importance of cell type when performing or comparing proteomics studies. Of these 43 proteins, 15 have been directly linked to LSD1 function and/or the CoREST complex, including all three CoREST family members, HDAC2, PHF21A, ZMYM2/3, GSE1 and CTBP1. In addition, more recently identified partners such as, SFMBT1, which forms a complex with LSD1–CoREST to repress gene targets, including cell cycle dependent repression of histone genes, were also identified using BioID.^62,63^ Among the extended group are LIN9 and MYBL2, part of the MMB complex described in ES cells above, inferring a functional link between MMB and LSD1 in multiple cell types.

Our data, and that from many other studies^64^ suggest BioID is an excellent method for examining protein–protein associations in cells enabling sampling of the proteome in a temporal fashion. Biotin ligation methods should prove to be a powerful tool for examining the complex association of proteins in cells, in particular for the large multi-protein complexes that regulate chromatin structure and gene expression. Our application of BioID in this study has identified a definitive network of LSD1 and CoREST complex associated proteins in three different cell types that should be a valuable resource for the field as we seek to address their fundamental roles during development and assess their potential as therapeutic targets.

## Materials and methods

### Cell culture

All ES cell experiments used stably transfected *Lsd1*^*lox/Δ3*^ cells^32^ cultured as described in M15 + leukemia inhibitory factor (LIF) media as described previously:^65^ 500 mL Knockout Dulbecco's Modified Eagle Medium (DMEM) (GIBCO; 10829-018), 90 mL Foetal Bovine Serum (FBS, Seralab; EU 000F), 6 mL Penicillin/Streptomycin/Glutamine-100× (GIBCO; 10378-016), 600 μL 50 mM β-mercaptoethanol (Fisher Scientific; 125472500), 25 μL LIF (prepared in house). To induce *Lsd1* deletion cells were cultured with 4-hydroxytamoxifen (OHT) as described previously.^3^ HEK293T cells were cultured in M10 media: 500 mL DMEM (GIBCO; 41963-039), 54 mL FBS, 6 mL Penicillin/Streptomycin/Glutamine-100× (GIBCO; 10378-016). To form embryoid bodies (EBs), ES cells were resuspended in differentiation media: 500 mL DMEM/F-12 (1 : 1) (GIBCO; 11320-003), 56.2 mL FBS (Seralab; EU 000F), 6 mL Penicillin/Streptomycin/Glutamine-100× (GIBCO; 10378-016), 600 μL β-mercaptoethanol (1000×) at 5 × 10^3^ cells per mL. 500 cells (100 μL) were plated in each well of low attachment-round bottomed 96 well plates (Corning;7007). EBs were imaged each day using a standard phase-contrast microscope with the size of individual EBs calculated using Nikon Imaging Software. For retinoic acid (RA) differentiation ES cells were plated down on pre-gelatinised 15 cm culture plates in M15 + LIF media. After 24 hours M15 + LIF media was replaced with the differentiation media described above supplemented with 1 μM RA. Cells were harvested after 0, 24 and 66 hours.

### Lipofectamine 2000 transfections

The cloning of all constructs used in the study was performed by the PROTEX service at the University of Leicester. For transient transfection of HEK293T cells on 10 cm plates 10 μg of plasmid DNA was incubated with 500 μL of Opti-MEM (GIBCO; 31985062), whilst 10 μL of Lipofectamine 2000 (Thermo Fisher; 11668019) was incubated with 500 μL of Opti-MEM separately for 5 min at room temperature. The DNA and Lipofectamine were mixed and incubated for 20 min before being added drop-wise to cells. For experiments in ES cells stable rescues were created. 5 × 10^5^*Lsd1*^*lox/Δ3*^ cells were dually transfected with *piggyBac* insertion and transposase vectors. 2.5 μg of each vector was incubated in the same 100 μL of Opti-MEM while 6 μL of Lipofectamine was incubated with a separate 100 μL of Opti-MEM at room temperature for 5 min. The DNA and lipofectamine were then mixed and incubated for a further 20 min before being added drop-wise to cells. 24 hours after transfection 5 × 10^3^ cells were plated on to 10 cm culture plates. Cells were treated with 200 μg mL^−1^ G418 (GIBCO; 10131027) for 10 days, single colonies were then picked on to 96 well round bottomed culture plates containing 50 μL of TrypLE (GIBCO; 12604021). The TrypLE suspension was then transferred on to a pre-gelatinised 96 well flat-bottomed plate containing 150 μL of M15 + LIF media. Cells were expanded before use in subsequent experiments.

### Immunoblotting

Cells grown to ∼80% confluence were harvested for protein extraction through washing in PBS and scraping in 1 mL PBS prior to centrifugation at 1100 rpm for 3 minutes to pellet cells. Cell pellets were resuspended in approximately 2–3× packed cell volume (PCV) of protein lysis buffer. Lysates were incubated on ice for 20 minutes and centrifuged at 14 000 rpm, 4 °C for 15 minutes to pellet cellular debris. Supernatants, containing the whole cell protein extract, were transferred to new eppendorfs and protein concentrations were determined with a Bradford assay. For extraction of histones, whole cell protein extractions were performed as described above. Following centrifugation and removal of supernatant, pellets were resuspended in 0.4 N H_2_SO_4_ (equal volume to volume of protein lysis buffer used). Samples were rotated at 4 °C for 24 hours prior to centrifugation at 14 000 rpm, 4 °C for 15 minutes to pellet cellular debris. Supernatants containing histone proteins were transferred to new eppendorfs and stored at −80 °C. The primary and secondary antibodies used are shown in Table T4 (ESI†). For blots used to probe biotinylation, a streptavidin infrared dye was used.

### BioID and mass spectrometry analysis

BirA* constructs were generated and transfected in to cells as described above. Where transient transfections were used, two 10 cm plates of HEK293T cells were transfected and 24 hours later 50 μM biotin was added to the culture media for 18 hours prior to harvesting. Stably transfected *Lsd1*^*lox/Δ3*^ ES cells expressing either BirA*-LSD1 or BirA*-NLS (generated as described above) were plated on to 15cm culture plates 24 hours prior to being treated with 50 μM biotin for 18 hours before harvesting. Cells were harvested by scraping in to 2 mL of PBS before pelleting through centrifugation at 1100 rpm for 3 min. Pellets were lysed in 1.5 mL of BioID lysis buffer (50 mM Tris–HCl pH 8.0, 150 mM NaCl, 0.5% NP-40, 0.5% Triton-X100, 0.1% SDS), and rotated at 4 °C for 20 min. Samples were then sonicated for 4 min (30 seconds on/30 seconds off), using the Bioruptor Pico Sonication device. Samples were cleared by centrifugation at 14 000 rpm at 4 °C for 15 min. Extracts were then added to 60 μL of Streptavidin agarose beads (Novagen; 69203-3) that had been washed 3 times in BioID lysis buffer. Extracts were incubated with the beads overnight whilst rotating at 4 °C. Beads were washed for 10 min in Wash Buffer 1 (2% SDS in ddH_2_O), Wash Buffer 2 (deoxycholate 0.2%, Triton X-100 1%, NaCl 500 mM, EDTA 1 mM, HEPES pH 7.5 50 mM), Wash Buffer 3 (Tris pH 8 10 mM, LiCl 250 mM, NP-40 0.5%, deoxycholate 0.5%, Triton X-100 1%, NaCl 500 mM, EDTA 1 mM), then twice in Wash Buffer 4 (Tris pH 7.4 50 mM, NaCl 50 mM). Streptavidin bead IPs were washed twice in 50 mM ammonium bicarbonate, then resuspended in 200 μL of ammonium bicarbonate, reduced with 10 mM tri(2-carboxyethyl) phosphine hydrochloride (TCEP) and alkylated with 20 mM Iodoacetamide in the dark at 37 °C, for 30 min. 1 μg of trypsin (Pierce, MS grade) was added to each sample and incubated whilst shaking overnight at 25 °C. Following trypsin digestion, streptavidin beads were pelleted and supernatants were acidified by addition of trifluroacetic acid to 0.4% and desalted using C18 spin columns (ThermoFisher) according to manufacturer's protocol. Samples were re-suspended in 40 μL of 0.5% formic acid and 18 μL was analysed by nanoflow LC-MS/MS using an Orbitrap Elite (Thermo Fisher) hybrid mass spectrometer equipped with a nanospray source, coupled to an Ultimate RSLCnano LC System (Dionex). The system was controlled by Xcalibur 3.0.63 (Thermo Fisher) and DCMSLink (Dionex). Peptides were desalted on-line using an Acclaim PepMap 100 C18 nano/capillary BioLC, 100A nanoViper 20 mm × 75 μm I.D. particle size 3 μm (Fisher Scientific) at a flow rate of 5 μL min^−1^ and then separated using a 125 min gradient from 5 to 35% buffer B (0.5% formic acid in 80% acetonitrile) on an EASY-Spray column, 50 cm × 50 μm ID, PepMap C18, 2 μm particles, 100 Å pore size (Fisher Scientific) at a flow rate of 0.25 μL min^−1^. The Orbitrap Elite was operated with a cycle of one MS (in the Orbitrap) acquired at a resolution of 60 000 at *m*/*z* 400, with the top 20 most abundant multiply charged (2+ and higher) ions in a given chromatographic window subjected to MS/MS fragmentation in the linear ion trap. An FTMS target value of 1 × 10^6^ and an ion trap MS^*n*^ target value of 1 × 10^4^ were used with the lock mass (445.120025) enabled. Maximum FTMS scan accumulation time of 500 ms and maximum ion trap MS^*n*^ scan accumulation time of 100 ms were used. Dynamic exclusion was enabled with a repeat duration of 45 s with an exclusion list of 500 and an exclusion duration of 30 s.

### Mass spectrometry data analysis

All raw mass spectrometry data were analysed with MaxQuant version 1.5.6 44. Data were searched against a human UniProt sequence database (June 2015) using the following search parameters: digestion set to Trypsin/P with a maximum of 2 missed cleavages, methionine oxidation and N-terminal protein acetylation as variable modifications, cysteine carbamidomethylation as a fixed modification, match between runs enabled with a match time window of 0.7 min and a 20 min alignment time window, label-free quantification enabled with a minimum ratio count of 2, minimum number of neighbours of 3 and an average number of neighbours of 6. A first search precursor tolerance of 20 ppm and a main search precursor tolerance of 4.5 ppm was used for FTMS scans and a 0.5 Da tolerance for ITMS scans. A protein FDR of 0.01 and a peptide FDR of 0.01 were used for identification level cut-offs. Protein group files generated by MaxQuant were loaded into Perseus version 1.5.5.3.^66^ The matrix was filtered to remove all proteins that were potential contaminants, only identified by site and reverse sequences. The LFQ intensities were then transformed by log_2_(*x*), and individual LFQ columns were grouped by experiment. Proteins were filtered to keep only those that had a minimum of 3 valid LFQ values in at least one group. The distribution of LFQ intensities of identified proteins was checked to ensure standard distribution for each individual replicate. Missing values were randomly imputed with a width of 0.3 and downshift of 1.8 from the standard deviation. In order to identify quantitatively enriched proteins between groups two-sided Student's *t*-tests were performed with a permutation-based FDR calculation (FDR = 0.05). Two levels of stringency for the Student's *t*-test were utilised based on the *S*0 parameter in Perseus which controls the relative importance of a *t*-test *p* value and difference between the means; *S*0 = 1, a lower stringency level, and *S*0 = 2 a higher stringency level. All subsequent analysis was performed on proteins significantly enriched in the higher stringency analysis. Gene ontology and interaction network visualisation was performed using Cytoscape (version 3.6.0).^54^

### Experimental design and statistical rationale

For the BioID experiments in HEK293T cells transient transfections were carried out in triplicate (BirA*-NLS, BirA*-PHF21A, BirA*-CoREST) or quadruplicate (BirA*-LSD1, HDAC1-BirA*), with 2 × 10 cm plates used per replicate. For ES cell experiments, four clones stably expressing either BirA*-LSD1 or BirA*-NLS were seeded on to 15 cm plates (1 × 15 cm plate per sample). The use of at least three biological replicates allowed for statistical determination of which proteins in our dataset were significantly more biotinylated by the fusion protein of interest, than the BirA*-NLS control. BirA*-NLS was used as a control in all experiments as the LSD1–CoREST complex which is predominantly localised to the nucleus. Therefore, BirA*-NLS allowed us to determine which proteins were incidentally biotinylated rather than being in close proximity to the protein of interest. Student's *t*-tests were used identify significantly enriched proteins using a permutation-based FDR calculation (FDR = 0.05). Student's *t*-tests were performed using Perseus which utilises the *S*0 parameter to weight the difference between means, all subsequent analysis was based on proteins that were significantly enriched using *S*0 = 2 which is the higher stringency level of analysis.

## Data availability

The mass spectrometry data have been deposited to the ProteomeXchange Consortium *via* the PRIDE (https://www.ebi.ac.uk/pride/archive/) partner repository with the dataset identifier PXD017344.

## Author contributions

Conception and design of the study: CEB, MOC and SMC. Data acquisition and analysis: CEB, MOC and SMC. The manuscript was written and edited by CEB, DAE, MB, MOC and SMC.

## Conflicts of interest

There are no conflicts to declare.

## Supplementary Material

MO-018-D1MO00236H-s001

MO-018-D1MO00236H-s002

MO-018-D1MO00236H-s003

MO-018-D1MO00236H-s004

MO-018-D1MO00236H-s005
