# Supplementary material for: Proximity-dependent biotin identification (BioID) reveals a dynamic LSD1–CoREST interactome during embryonic stem cell differentiation
Source: Mol Omics. 2021 Oct 14;18(1):31–44. doi: 10.1039/d1mo00236h (PMC8763317; doi:10.1039/d1mo00236h)
Supplement: MO-018-D1MO00236H-s001 [file MO-018-D1MO00236H-s001.pdf]

Electronic Supplementary Material (ESI) for Molecular Omics.
This journal is © The Royal Society of Chemistry 2021

Supplementary information

Proximity-dependent biotin identification (BioID) reveals a dynamic LSD1-CoREST interactome during embryonic stem cell differentiation

Claire E Barnes[1], David A English[1], Megan Broderick[1], Mark O Collins[2,3] and Shaun M Cowley[1,*]

[1] Department of Molecular and Cell biology, Henry Wellcome Building, University of Leicester, Leicester, LE1 9HN, United Kingdom

[2] School of Biosciences, the University of Sheffield, Western Bank, Sheffield S10 2TN

[3] Faculty of Science Mass Spectrometry Centre, University of Sheffield, Brook Hill Road, Sheffield, S3 7HF, United Kingdom.

*To whom correspondence should be addressed

List of supplementary data:

Supplementary Figure 1 – Expression of BirA*-tagged CoREST complex members permits biotinylation of proximal proteins.

Supplemental Figure 2 – Western blot for the indicated proteins using whole cell protein extracts isolated during sampling for BioID in ES cells.

Supplementary Table 1 – 293T BioID dataset

Supplementary Table 2 – ESC LSD1 BioID dataset

Supplementary Table 3 – 293T and ESC overlapping targets

Supplementary Table 4 – Antibodies used in this study

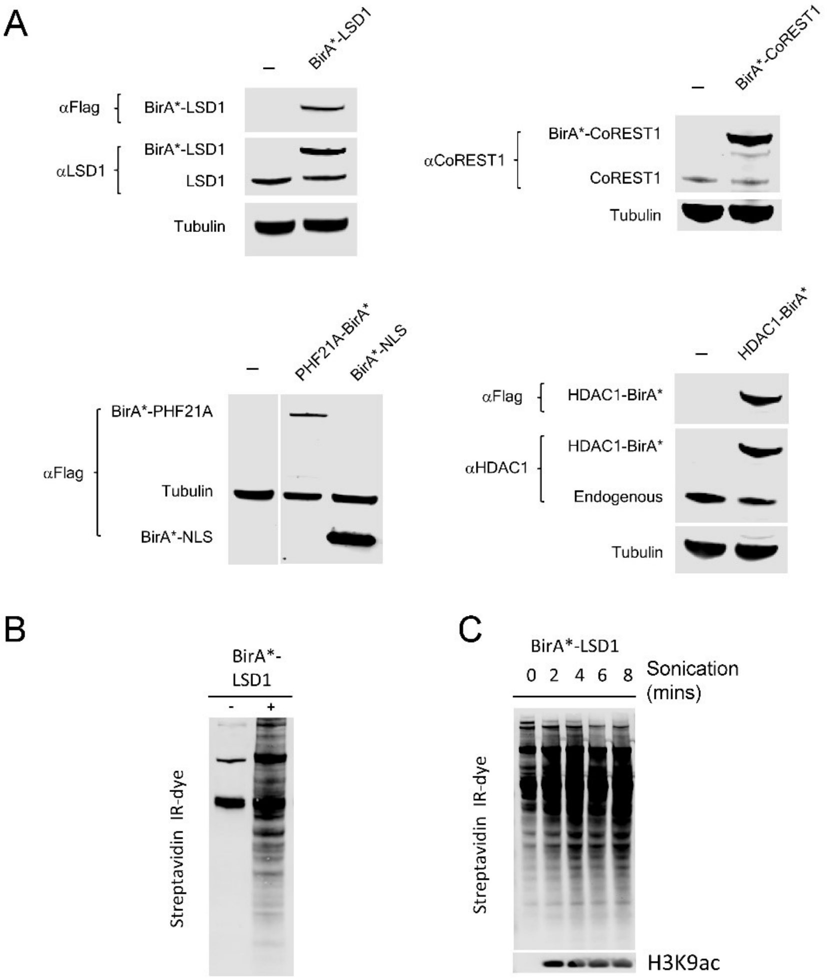

Supplementary Figure 1. Expression of BirA*-tagged CoREST complex members permits biotinylation of proximal proteins. **A**, western blots for the indicted proteins using either anti-FLAG, or antibodies specific to the protein of interest, as indicated. **B**, Biotinylated proteins, with and without the addition of exogenous biotin, detected using streptavidin conjugated to an infra-red^{800} dye. **C**, Whole cell protein extracts were made from pellets sonicated for the indicated times, with biotinylated proteins detected using streptavidin conjugated to an infra-red^{800} dye.

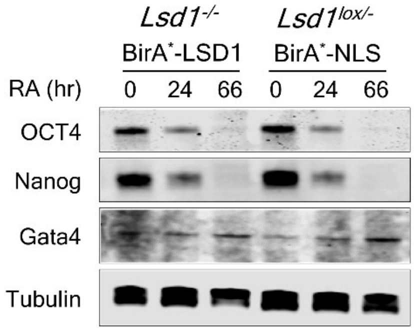

Supplemental Figure 2. Western blot for the indicated proteins using whole cell protein extracts isolated during sampling for BioID in ES cells. The time-course of retinoic acid (RA) treatment is indicated. Proteins were extracted from either control (*Lsd1*$^{Lox/-}$) and *Lsd1* knockout (*Lsd1*$^{-/-}$) ES cells.
