# Supplementary material for: Proximity-dependent biotin identification (BioID) reveals a dynamic LSD1–CoREST interactome during embryonic stem cell differentiation
Source: Mol Omics. 2021 Oct 14;18(1):31–44. doi: 10.1039/d1mo00236h (PMC8763317; doi:10.1039/d1mo00236h)
Supplement: MO-018-D1MO00236H-s005 [file MO-018-D1MO00236H-s005.pdf]

Electronic Supplementary Material (ESI) for Molecular Omics.
This journal is © The Royal Society of Chemistry 2021

Supplementary Table 4.

Primary antibodies used for Immunoblotting.

Antibody	Source	Dilution	Company	Product No.
α-tubulin	Mouse	1:15000	Sigma	T5168
CoREST1	Mouse	1:1000	Millipore	MABN486
FLAG	Mouse	1:2500	Sigma	F1804
Gata4	Rabbit	1:2000	Abcam	Ab134057
H3K9Ac	Rabbit	1:1000	Millipore	06-942
HDAC1	Rabbit	1:2000	Abcam	Ab109411
LSD1	Rabbit	1:10000	Abcam	Ab129195
NANOG	Rabbit	1:2000	Bethyl	A300-397A
OCT4	Mouse	1:500	Santa Cruz	Sc-5279

Secondary antibodies used for Immunoblotting.

Antibody	Source	Dilution	Company	Product No.
IRDye 800CW-Mouse	Goat anti-mouse IgG	1:10000	LICOR	926-32210
IRDye 680CW-Rabbit	Donkey anti-Rabbit	1:10000	LICOR	926-68023
Streptavidin IRDye 800CW		1:5000	LICOR	926-32230
